# Role of galactomannan determinations in bronchoalveolar lavage fluid samples from critically ill patients with chronic obstructive pulmonary disease for the diagnosis of invasive pulmonary aspergillosis: a prospective study

**DOI:** 10.1186/cc11443

**Published:** 2012-07-27

**Authors:** Hangyong He, Lin Ding, Bing Sun, Fang Li, Qingyuan Zhan

**Affiliations:** 1 Department of Respiratory and Critical Care Medicine, Beijing Institute of Respiratory Medicine, Beijing Key Laboratory of Respiratory and Pulmonary Circulation, Beijing Chao-Yang Hospital, Capital Medical University, No. 8 Gongren Tiyuchang Nanlu, Chaoyang District, Beijing (100020), China; 2 Department of Infectious Diseases and Clinical Microbiology, Beijing Institute of Respiratory Medicine, Beijing Key Laboratory of Respiratory and Pulmonary Circulation, Beijing Chao-Yang Hospital, Capital Medical University, No. 8 Gongren Tiyuchang Nanlu, Chaoyang District, Beijing (100020), China

**Keywords:** bronchoalveolar lavage, invasive pulmonary aspergillosis, galactomannan, intensive care unit, chronic obstructive pulmonary disease

## Abstract

**Introduction:**

Critically ill chronic obstructive pulmonary disease (COPD) patients are at particular risk of invasive pulmonary aspergillosis (IPA). Our aims were to determine whether bronchoalveolar lavage fluid (BALF) galactomannan (GM) has a higher sensitivity and specificity than serum GM or lower respiratory tract (LRT) sample culture. Furthermore, we aimed to investigate what the optimal cut-off value would be for BALF GM.

**Methods:**

In this prospective single-center study, BALF and serum samples were collected from critically ill COPD patients on the first day of their intensive care unit admission.

**Results:**

Of 50 critically ill COPD patients admitted, BALF and serum samples were collected in 34 patients. According to the receiver operating characteristics (ROC) curve, an optical density (OD) ratio of 0.8 was chosen as the cut-off value for GM in BALF. Compared to serum GM and LRT *Aspergillus *isolation, BALF GM yield a better sensitivity, specificity, positive and negative predictive values of 88.9%, 100%, 100% and 94.4%, respectively. Areas under the ROC curve were 0.912 (95%CI, 0.733 to 0.985) for BALF GM, and 0.879 (95%CI, 0.691 to 0.972) for serum GM results from the first day of ICU admission. Pairwise comparison of ROC curves showed *P *= 0.738. The OD ratio of BALF GM in IPA patients were significantly higher than those of non-IPA patients (2.88 ± 2.09 versus 0.49 ± 0.19, *P *= 0.009), and the OD ratio of BALF GM was significantly higher than serum GM in IPA patients (2.88 ± 2.09 versus 0.87 ± 0.47, *P *= 0.023). Positive BALF GM was seen earlier than LRT secretion culture (1 day versus 3.8 days).

**Conclusions:**

Compared to serum GM and LRT *Aspergillus *isolation, BALF GM seems to have a better sensitivity in the diagnosis of IPA in critically ill COPD patients. The ROC curve suggests a possible cut-off value of 0.8 for GM from BALF specimens in critically ill COPD patients.

## Introduction

Invasive pulmonary aspergillosis (IPA) is an important cause of mortality in imunocompromised populations, such as hematological and solid-organ transplant patients. In recent years, chronic obstructive pulmonary disease (COPD) patients, especially those who are critically ill and are admitted to an ICU, are being increasingly recognized as belonging to a population at particular risk of IPA. Timely diagnosis of IPA in this population is challenging due to a lack of specific signs and symptoms, which contributes to a high mortality of 67% to 100% [1-11].

The Platelia *Aspergillus *enzyme immunoassay has being used internationally to detect serum and bronchoalveolar lavage fluid (BALF) galactomannan (GM) for early diagnosis of IPA. In the immunocompromised population with IPA, serum and BALF GM has been evaluated extensively [12-15], and is proposed by the European Organization for Research and Treatment of Cancer/Mycoses Study Group (EORTC/MSG) Consensus Group as one of the mycological criteria in the probable IPA diagnosis for this population [16].

The value of serum GM in COPD has been investigated in several studies [9,10,17]. As opposed to immunocompromised populations, these studies demonstrated a lower sensitivity of only about 30% to 58% in COPD. The low sensitivity of the galactomannan determination in serum samples from non-neutropenic patients may be a consequence of two factors: the clearance of GM by circulating neutrophils and the less angioinvasive forms of *Aspergillus *infection in these patients that impair the access of GM to the blood stream [18-20]. The sensitivity of BALF GM may be higher than that from serum because of the high fungal burden in the bronchial tree of patients with IPA. BALF GM has been evaluated extensively in neutropenic patients with high sensitivity. However, data on BALF GM for IPA diagnosis in critically ill COPD patients are limited [21] and, so far, no cut-off value for BALF GM has been established for the diagnosis of IPA in this population. The aim of this prospective single center study is to determine whether BALF GM has a higher sensitivity and specificity than serum GM or lower respiratory tract (LRT) culture. Furthermore, we aimed to investigate what the optimal cut-off value would be for BALF GM.

## Materials and methods

### Study population and data collection

In our study, all of the patients were admitted to a respiratory intensive care unit (RICU) because of respiratory failure from February 2009 to December 2009. These patients were older than 18 years and had been diagnosed with severe COPD, stage III or IV according to the Global Initiative for Chronic Obstructive Lung Disease (GOLD). Patients were excluded for: (i) concurrent solid organ tumor; (ii) concurrent hematological system diseases; (iii) organ transplantation; (iv) long-term use of glucocorticoids for reasons other than COPD; and (v) receiving radiation therapy or immunosuppressive drugs.

The following information was stored in a data file: patients' characteristics, including age, sex, medical history, reasons for ICU admission; the risk factors for IPA, including the use of immunosuppressive drugs (steroids and others), antibiotics and their nutrition [8,22-24]; the presence of recent exacerbation of dyspnea and rales, standard ICU laboratory and microbiology findings; and outcome and cause of death. Beijing Chao-yang Hostipital IRB approved this study and informed consent was requested from patients or their next of kin before they were included in the study.

### Collection and processing of clinical samples

BALF and serum sample collection for GM detection was done on the first day after patients' admission if feasible, depending on the patients' situations.

Fiberoptic bronchoscopy with bronchoalveolar lavage (20 ml for three times) was performed by experienced doctors, if feasible. The sampling area was selected based on the infiltration location on a chest radiograph. If there were no apparent lesions, the bronchoscope would be wedged into a segmental airway of the right middle lobe. Lavage samples were submitted for direct microscopic examination and microbiological culture. A vortex was done for the remaining pooled BALF and the supernatant was stored for GM detection. Five milliliter serum was collected with a non-anticoagulation tube and was processed the same way as BALF for the detection of galactomannan.

A sandwich ELISA assay for GM detection (Platelia *Aspergillus*; Sanofi Diagnostics Pasteur, Marnes-La-Coquette, France) was performed according to the manufacturer's instructions. An optical density (OD) ratio of 0.5 or greater for serum GM was considered positive [4].

LRT samples (including sputum, endotracheal aspiration) of all patients included in this study were taken once a day for the first three days of their ICU stay. LRT samples were collected again once per week if the patient remained in the ICU for more than seven days. All LRT specimens and BALF samples were cultured on conventional media, including sheep-blood agar and chocolate agar. At the same time, all LRT specimens were cultured on Sabouraud dextrose agar. Samples and specimens were incubated at 25°C and 37°C, respectively, for 7 to 14 days. When spore growth was suboptimal on the routine media, LRT samples were further cultured on potato dextrose agar for a better conidial production. *Aspergillus *isolates were identified by using standard morphologic procedures, including colony morphology, growth velocity, color, morphology of hyphae, and characteristics of hyphae and spores under microscopy.

### Case definitions

Each of the patients was classified based on the case definition proposed by Bulpa *et al. *[4]. Thus, cases were diagnosed and classified as proven, probable, possible IPA or *Aspergillus *colonization, and non-IPA. Proven cases required histopathological or cytopathological confirmation. Probable cases required both host factors (COPD patient, had recent exacerbation of dyspnea and suggestive chest imaging, and typically had poor response to conventional treatment) and microbiological factors (isolation of *Aspergillus *in the LRT samples or two consecutive positive serum GM tests). Possible cases required host factors, but without microbiological proof. Colonization was defined as asymptomatic and isolation of *Aspergillus *in LRT samples.

### Statistical analysis

Patients were divided into IPA (proven, probable and possible cases) and non-IPA patients (colonization and control cases). For further analysis, possible and colonization patients were excluded from IPA and non-IPA patients. Using this standard, the ability to predict infection in critically ill COPD patients on the basis of BALF GM with a different cut-off value (OD ratio = 0.8 and 0.5, respectively), serum GM and LRT *Aspergillus *isolation was assessed with respect to the sensitivity, specificity, positive predictive value, and negative predictive value of these tests. Receiver operating characteristics (ROC) curves were generated and compared between serum GM and BALF GM to illustrate the performance of these two methods. The performance of serum GM on the first day of ICU admission was used in the comparison between serum GM and BALF GM. Normally distributed continuous variables were expressed as mean ± SD and compared with a *t *test. *P *values < 0.05 were considered significant. All analyses were carried out with the use of SPSS software for Windows (release 11.5).

## Results

### Patient characteristics and outcomes

From February 2009 to December 2009, in total, 298 patients were admitted to our ICU because of respiratory failure. Of these patients, 60 COPD patients were admitted to our ICU and 50 COPD patients who met the inclusion criteria were enrolled. Sixteen patients did not need tracheal intubation and invasive mechanical ventilation, and bronchoscopy was not performed and BALF samples were not collected in these patients. Thirty-four BALF samples were collected, twenty-one were from male and 13 were from female patients. According to the criteria for IPA, the 34 patients with a BALF sample collected were classified as probable (*n *= 9), possible (*n *= 6), colonization (*n *= 2) and non-IPA (*n *= 17). Of the nine probable IPA patients, four patients used a broad-spectrum antibiotic for more than 20 days and the other five patients received one for more than seven days. Despite antifungal treatments after IPA was diagnosed, all of these nine patients died (Table 1). Two of the six possible IPA patients died and the other four patients recovered. Two patients with *Aspergillus *isolation were considered as colonization because their conditions were improved with no pulmonary lesions revealed on their chest radiography and no antifungal treatment. Fifteen non-IPA patients survived. The other two non-IPA patients died of multiple organ failure and respiratory failure, respectively.

**Table 1:** Characteristics of probable IPA in critically ill COPD patients (*n *= 9)

	Age (years)	Risk factors	The length from symptoms to ICU admission (day)	APACHE II scores	Samples of fungal culture	*Aspergillus *species	Serum GM			BALF GM	The time of antifungal treatment after ICU admission	Cause of death
							Day 1	Day 4	Day 7			
1	76	Antibiotics steroid	13	14	ETA	none	0.298	0.503	1.321	4.508	Day 2	MOF
2	52	Antibiotics steroid	20	15	ETA	*A. fumigatus*	0.296	0.484	-	0.322	Day 3	RF
3	74	Antibiotics steroid	11	15	ETA BALF	*A. fumigatus *&*A. flavus*	0.626	3.170	-	6.374	Day 2	MOF, SS
4	49	Antibiotics	24	15	ETA	none	1.606	1.302	-	1.098	Day 1	MOF
5	87	Antibiotics	17	7	ETA	none	0.867	2.499	-	1.510	Day 5	MOF, SS
6	71	Antibiotics steroid	7	10	ETA	*A. fumigatus*	0.562	0.620	-	0.850	Day 6	RF
7	81	Antibiotics steroid	30	15	ETA	*A. fumigatus *&*A. versicolor*	1.081	2.032	-	2.609	Day 2	MOF
8	59	Antibiotics	60	14	ETA	*A. fumigatus*	1.432	-	-	4.057	Day 1	MOF, SS
9	65	Antibiotics steroid	10	18	ETA BALF	*A. fumigatus *&*A. fiavus*	1.072	0.533	-	4.619	Day 1	MOF

APACHE II, Acute Physiology and Chronic Health Evaluation II; BALF, bronchoalveolar lavage fluid; GM, galactomannan, MOF, multiple organ failure; RF, renal failure; SS, septic shock.

The nine critically ill probable IPA patients with COPD are described in Table 1. All of the nine probable IPA patients had progressive dyspnea, eight patients had a fever over 38°C and six patients still had fever 72 hours after they received broad spectrum {here should be no blank lines between "spectrum" and "antibiotics"}antibiotics. Chest pain occurred in only one IPA patient and no hemoptysis was observed. Congestion and edema of the bronchial mucosa were observed under bronchoscopy, and infiltrates were seen on the chest radiography in all IPA patients. Five patients had a high resolution computed tomography (HRCT) scan, and only one patient showed cavity. Bacterial cultures of BALF or bronchial secretions showed *Acinetobacter baumanii*, *Escherichia coli *and *Burkholderia yabunchi *in four, one and one IPA patients, respectively. Anti-bacterial therapy was given to these patients according to the culture results and the main cause of respiratory infection was considered to be the *Aspergillus *species.

### BALF GM, serum GM, and LRT sample culture

Levels of BALF and serum GM of IPA patients are shown in Table 1. Eight IPA patients had an OD ratio of BALF GM of more than 0.85, while only one IPA patient had a BALF GM less than 0.5. The BALF GM levels in IPA patients were significantly higher than in their serum (OD ratio of 2.88 ± 2.09 versus 0.87 ± 0.47, *P *= 0.023). BALF GM levels were significantly higher in IPA patients compared with non-IPA patients (OD ratio of 2.88 ± 2.09 versus 0.49 ± 0.19, *P *= 0.009). Only two patients received piperacillin/tazobactam treatment before and during their ICU stay. One patient had a BALF GM of 0.693, but with a negative serum GM of 0.166. The BALF and serum GM were 0.322 and 0.283 for the other patient. No association was observed between antibiotic use and GM levels in either serum or BALF samples.

The sensitivity, specificity, and the positive and negative predictive values of BALF GM, serum GM and LRT *Aspergillus *isolation for the patients with true infection (probable cases) and those without infection (non-IPA), and for all patients (including probable, possible, colonization and non-IPA patients) are shown in Table 2 and Table 3 respectively. Compared to serum GM and LRT *Aspergillus *isolation, BALF GM showed a better sensitivity and negative predictive values, a same specificity and positive predictive values of 88.9%, 94.4%, 100% and 100% for probable IPA in critically ill COPD patients, respectively. However, the confidence intervals of the sensitivity estimates overlap between BALF, serum and LRT.

**Table 2:** Results of BALF and serum GM detection and isolation of *Aspergillus *from LRT in critically ill COPD patients.

	BALF GM ( 95%CI) (cut-off 0.8)	BALF GM ( 95%CI) (cut-off 0.5)	Serum GM ( 95%CI) (cut-off 0.5)	Isolation of *Aspergillus *from LRT ( 95%CI)
Sensitivity (%)	88.9 (76.8 to 101)	88.9 (76.8 to 101)	77.8 (61.8 to 93.8)	66.7 (48.6 to 84.8)
Specificity (%)	100 (100 to 100)	47.1 (27.9 to 66.3)	100 (100 to 100)	100 (100 to 100)
PPV (%)	100 (100 to 100)	47.1 (27.9 to 66.3)	100 (100 to 100)	100 (100 to 100)
NPV (%)	94.4 (85.6 to 103.2)	88.9 (76.8 to 101)	89.5 (77.7 to 101.3)	85.0 (71.3 to 98.7)
TCR (%)	96.1 (88.7 to 103.5)	61.5 (42.8 to 80.2)	92.3 (82.1 to 102.5)	88.5 (76.2 to 100.8)

For probable IPA and control patients (*n *= 26).

BALF, bronchoalveolar lavage fluid; COPD, chronic obstructive pulmonary disease; GM, galactomannan; IPA, invasive pulmonary aspergillosis; LRT, lower respiratory tract; NPV, negative predictive value; PPV, positive predictive value; TCR, total consistent rate.

**Table 3:** BALF, serum GM and *Aspergillus *isolation from LRT.

	BALF GM ( 95%CI) (cut-off 0.8)	BALF GM ( 95%CI) (cut-off 0.5)	Serum GM ( 95%CI) (cut-off 0.5)	Isolation of *Aspergillus *from LRT ( 95%CI)
Sensitivity (%)	66.7 (48.6 to 84.8)	73.3 (56.3 to 90.3)	53.3 (34.1 to 72.5)	46.7 (27.5 to 65.9)
Specificity (%)	100 (100 to 100)	52.6 (33.4 to 71.8)	100 (100 to 100)	89.5 (77.7 to 101.3)
PPV (%)	100 (100 to 100)	55.0 (35.9 to 74.1)	100 (100 to 100)	77.8 (61.8 to 93.8)
NPV (%)	79.1 (63.5 to 94.7)	71.4 (54.0 to 88.8)	73.0 (55.9 to 90.1)	68.0 (50.1 to 85.9)
TCR (%)	85.3 (71.7 to 98.9)	61.8 (43.1 to 80.5)	79.4 (63.9 to 94.9)	70.6 (53.1 to 88.1)

For probable, possible IPA, colonization and control patients (*n *= 34).

BALF, bronchoalveolar lavage fluid; CI, confidence interval; COPD, chronic obstructive pulmonary disease; GM, galactomannan; IPA, invasive pulmonary aspergillosis; LRT, lower respiratory tract; NPV, negative predictive value; PPV, positive predictive value; TCR, total consistent rate.

**Figure 1:**
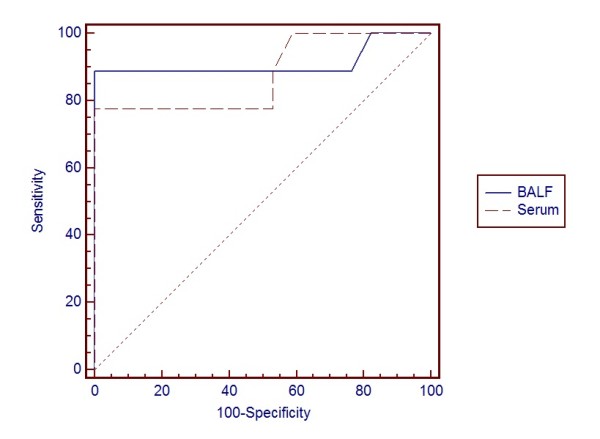
**For probable IPA and control patients (*n *= 26)**. Receiver operating characteristics (ROC) curve for galactomannan (GM) detection in bronchoalveolar lavage fluid (BALF) and serum graphing sensitivity (true positive results) versus 1-specificity (true negative results) using BALF GM and serum GM to define positivity. Areas under the ROC curve were 0.912 (95%CI, 0.733 to 0.985) for BALF GM and 0.879 (95%CI, 0.691 to 0.972) for serum GM result on the first day of ICU admission. Pairwise comparison of ROC curves showed *P *= 0.738. CI, confidence interval.

**Figure 2:**
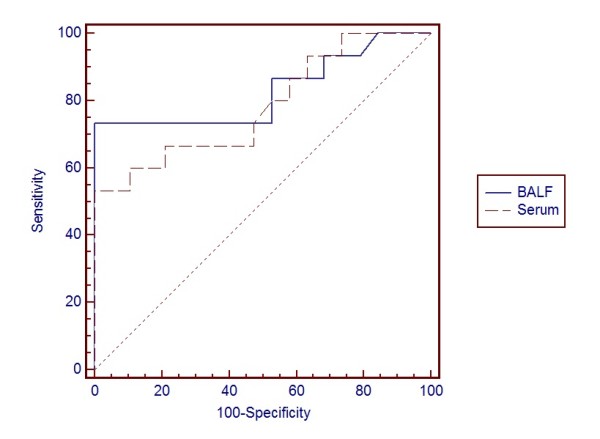
**For probable IPA, possible IPA, colonization and control patients (*n *= 34)**. Receiver operating characteristics (ROC) curve for galactomannan (GM) detection in bronchoalveolar lavage fluid (BALF) and serum graphing sensitivity (true positive results) versus 1-specificity (true negative results) using BALF GM and serum GM to define positivity. Areas under the ROC curve were 0.830 (95%CI, 0.662 to 0.936) for BALF GM and 0.784 (95%CI, 0.610 to 0.906) for serum GM result on the first day of ICU admission. Pairwise comparison of ROC curves showed *P*= 0.604. CI, confidence interval.

The ROC curves for BALF and serum GM for the patients with true infection (probable cases) and those without infection (non-IPA), and for all patients (including probable, possible, colonization and non-IPA patients) are shown in Figure 1 and 2. The ROC curve suggested an optimal cut-off value of 0.8 for the OD ratio of BALF GM. Comparisons of the area under the curve between BALF and serum were not statistically significant in either test.

For the 34 patients whose BALF were collected, mortality was significantly higher in patients whose OD ratios of BALF GM were more than 0.8 than in those patients whose BALF GM were less than 0.8 (80% versus 25%, *P *= 0.002).

The sensitivity of direct microscopy and culture of LRT specimens were 11% and 67% for probable IPA and control patients, respectively. In one IPA patient (Table 1 case 1), BALF GM showed positive three days before his serum GM turned positive. Positive BALF GM emerged significantly earlier than LRT sample culture (1 day versus 3.8 days).

## Discussion

Our study revealed that, compared to LRT *Aspergillus *isolation and serum GM, BALF GM might have a better sensitivity and a same specificity, and may improve the diagnosis of IPA in critically ill COPD patients.

When critically ill COPD patients admitted to ICU, are intubated and mechanically ventilated, LRT samples can be more easily collected. However, the sensitivity of *Aspergillus *culture and direct microscopic examination was only about 40% [25]. In addition, *Aspergillus *culture takes at least 24 to 72 hours and may delay the diagnosis of IPA. In our study, the sensitivity of direct microscopy and culture of LRT specimens were 11% and 67%, respectively, which is lower than serum and BALF GM.

Serum GM has been evaluated extensively in neutropenic patients with a sensitivity of 71% and a specificity of 89%. Furthermore, positive serum GM is proposed as a criterion for probable IPA by the EORTC/MSG Consensus Group [12-14,16]. However, for non-neutropenic patients, including COPD patients, some reports suggested a low sensitivity of serum GM for IPA [18,19]. Besides, several factors may cause false-positive results in serum GM determination, such as intravenous use of piperacillin/tazobactam, amoxicillin/clavulanic acid, albumin, and the contamination of water and food [26,27].

In COPD patients, because the structures and defense functions of the airways and lung parenchyma are damaged by underlying respiratory diseases, *Aspergillus *may colonize in these sites. During the early period of invasive aspergillosis, infection may be limited to the tracheobronchial region, presenting as *Aspergillus *tracheal-bronchitis. With corticosteroids and broad-spectrum antibiotics therapy, the infection could spread to the distal airways and lung parenchyma, presenting as IPA [10]. During the later stage, hyphae invade into the blood vessels, and galacotomannan is released into the circulation. GM from BALF may be increased earlier than that from serum because of the high fungal burden in the bronchial region in the early stage of IPA. A previous study indicated that in patients admitted to ICU, including critically ill COPD patients, when using a cutoff index of 0.5, the sensitivity and specificity of BALF GM was 88% and 87%, respectively, while the sensitivity of serum GM was only 42% [28]. Our study suggests that, on the first day of ICU admission, the average level of BALF GM was significantly higher than that of serum GM in COPD patients with IPA.

One of the main problems encountered in GM assay in BALF is that no standardized procedure for BALF collection is defined at present. In our study, we performed the bronchoalveolar lavage procedure used in a previous study for ICU patients [28]. The BALF was obtained after three successive injections of 20 mL of saline. This volume could be considered as a low volume, especially for the COPD patients. Due to their obstructive ventilatory disorder, removal of injected fluid could be frequently ineffective. However, the volume retrieved in our patients ranged from 24 mL to 42 mL, which was approximately 50% of the volume of the injection. The variation of the volume is important because the amount of fluid injected and retrieved may influence the final concentration of GM. Independent of this dilution factor, it is empirically known that a 'small' lavage explores the alveoli less deeply than a 'large' one. Therefore, this is a point likely to introduce discrepancies in studies focusing on this topic, and which volume and number of times the injection should be performed needs to be standardized in future studies.

The EORTC/MSG criteria for IPA were developed for patients with malignant disease and recipients of allogeneic hematopoietic stem cell and solid-organ transplants [16]. Due to the absence of data, the criteria do not apply to non-cancer populations. In 2007, Bulpa *et al*. proposed their definitions of IPA, specifically for COPD patients and isolation of *Aspergillus *[4] based on several case reports. The diagnoses for 36 patients (64%) were classified as proven in their study, which validated their criteria. Their criteria have been used as diagnostic criteria in several recent reports [17,29,30], and may be more appropriate for IPA in our study with the COPD population.

The frequency of IPA in this study was 15% (9/60), and higher than that in the recent paper by Aquino *et al*. on a similar patient population [31]. The incidence of IPA in COPD populations from whom the *Aspergillus *was recovered in culture was 16.1% to 22.1% [17]. This incidence was even higher, at 37%, for those COPD patients whose specimens yielded *Aspergillus *and who required admission to the ICU [17].

The current study has several limitations. First, since the collection and processing of BALF samples has not yet been standardized, we followed the method used in a previous study [28]. Second is the relatively small sample size. Third, serum GM and LRT *Aspergillus *isolation were included in the criteria to define IPA. This may have caused 'incorporation bias'. By incorporating the test(s) under evaluation in the reference standard (the IPA criteria), the sensitivity and specificity of these tests may look higher than they are in reality. Finally, although all probable IPA patients enrolled were non-survivors, post-mortem examinations were rejected by the family members of these patients and were not performed for a definitive diagnosis of invasive aspergillosis.

## Conclusions

Compared to serum GM and LRT *Aspergillus *isolation, BALF GM appears to have a higher sensitivity in the early diagnosis of IPA in critically ill COPD patients. The ROC curve suggests a possible cut off value of 0.8 for BALF specimens. Evidence-based prospective studies are needed for further evaluation of the usefulness and standardization of the procedure of BALF GM in critically ill COPD patients.

## Key messages

• Compared to serum GM and LRT culture, BALF GM appears to have an increased sensitivity in the diagnosis of IPA in critically ill COPD patients.

• A possible cut off value of OD ratio of 0.8 for GM from BALF specimens may be suggested for the diagnosis of IPA in critically ill COPD patients.

## Abbreviations

BALF: bronchoalveolar lavage fluid; COPD: chronic obstructive pulmonary disease; ELISA: enzyme-linked immunosorbent assay; EORTC/MSG: the European Organization for Research and Treatment of Cancer/Mycoses Study Group; GM: galacotomannan; IPA: invasive pulmonary aspergillosis; LRT: lower respiratory tract; OD: optical density; ROC: receiver operating characteristics.

## Competing interests

The authors declare that they have no competing interests.

## Authors' contributions

All authors made substantial contributions to conception and design, or acquisition of data, or analysis and interpretation of data; reviewed and approved the final manuscript; and contributed significantly to this study. HH and LD contributed equality to the work. QZ takes full responsibility for the integrity of the submission and publication, and was involved in study design. HH had full access to all the data in the study and takes responsibility for the integrity of the data and the accuracy of the data analysis, and was responsible for data verification, analysis and drafting of the manuscript. LD had full access to all the data in the study and takes responsibility for the integrity of the data and the accuracy of the data analysis. BS and FL were responsible for the microbiological examination and data collection.

## References

[B1] Lin SJ Schranz J Teutsch SM Aspergillosis case-fatality rate: systematic review of the literature Clin Infect Dis 2001 32 358 366 10.1086/318483 11170942

[B2] Ader F Nseir S Berre RL Leroy S Tillie-Leblond I Marquette C Durocher A Invasive pulmonary aspergillosis in chronic obstructive pulmonary disease: an emerging fungal pathogen Clin Microbiol Infect 2005 11 427 429 10.1111/j.1469-0691.2005.01143.x 15882191

[B3] Meersseman W Vandecasteele SJ Wilmer A Verbeken E Peetermans WE Wijngaerden EV Invasive aspergillosis in critically ill patients without malignancy Am J Respir Crit Care Med 2004 170 621 625 10.1164/rccm.200401-093OC 15229094

[B4] Bulpa P Dive A Sibille Y Invasive pulmonary aspergillosis in patients with chronic obstructive pulmonary disease Eur Respir J 2007 30 782 800 10.1183/09031936.00062206 17906086

[B5] Zhan Q He H Tong Z Li F Sun B Wang C Clinical features of invasive pulmonary aspergillosis in critically ill patients with chronic respiratory diseases Zhonghua Jie He He Hu Xi Za Zhi 2008 31 282 286 18846966

[B6] Muquim A Dial S Menzies D Invasive aspergillosis in patients with chronic obstructive pulmonary diseases Can Respir J 2005 12 199 204 16003456 10.1155/2005/676878

[B7] Dimopoulos G Piagnerelli M Berre J Eddafali B Salmon I Vincent JL Disseminated aspergillosis in intensive care unit patients: an autopsy study J Chemother 2003 15 71 75 12678418 10.1179/joc.2003.15.1.71

[B8] Samarakoon P Soubani A Invasive pulmonary aspergillosis in patients with COPD: a report of five cases and systematic review of the literature Chronic Respiratory Disease 2008 5 19 21 10.1177/1479972307085637 18303098

[B9] He H Ding L Chang S Li F Zhan Q Value of consecutive galactomannan determinations for the diagnosis and prognosis of invasive pulmonary aspergillosis in critically ill chronic obstructive pulmonary disease Med Mycol 2011 49 345 351 10.3109/13693786.2010.521523 20936913

[B10] He H Ding L Li F Zhan Q Clinical features of invasive bronchial-pulmonary aspergillosis in critically ill patients with chronic obstructive respiratory diseases: a prospective study Crit Care 2011 15 R5 10.1186/cc9402 21211008 PMC3222032

[B11] Segal BH Aspergillosis N Engl J Med 2009 360 1870 1884 10.1056/NEJMra0808853 19403905

[B12] Maertens J Eldere JV Verhaegen J Verbeken E Verschakelen J Boogaerts M Use of circulating galactomannan screening for early diagnosis of invasive aspergillosis in allogeneic stem cell transplant recipients J Infect Dis 2002 186 1297 1306 10.1086/343804 12402199

[B13] Maertens J Verhaegen J Lagrou K Van Eldere J Boogaerts M Screening for circulating galactomannan as a noninvasive diagnostic tool for invasive aspergillosis in prolonged neutropenic patients and stem cell transplantation recipients: a prospective validation Blood 2001 97 1604 1610 10.1182/blood.V97.6.1604 11238098

[B14] Pfeiffer C Fine J Safdar N Diagnosis of invasive aspergillosis using a galactomannan assay: a meta-analysis Clin Infect Dis 2006 42 1417 1427 10.1086/503427 16619154

[B15] Maertens J Maertens V Theunissen K Meersseman W Meersseman P Meers S Verbeken E Verhoef G Van Eldere J Lagrou K Bronchoalveolar lavage fluid galactomannan for the diagnosis of invasive pulmonary aspergillosis in patients with hematologic diseases Clin Infect Dis 2009 49 1688 1693 10.1086/647935 19886801

[B16] Pauw BD Walsh T Donnelly J Stevens D Edwards J Calandra T Pappas P Maertens J Lortholary O Kauffman C Denning D Patterson T Maschmeyer G Bille J Dismukes W Herbrecht R Hope W Kibbler C Kullberg B Marr K Munoz P Odds F Perfect J Restrepo A Ruhnke M Segal B Sobel J Sorrell T Viscoli C Wingard J Zaoutis T Bennett J EORTC/MSG Consensus Group Revised definitions of invasive fungal disease from the European Organization for Research and Treatment of Cancer/Invasive Fungal Infections Cooperative Group and the National Institute of Allergy and Infectious Diseases Mycoses Study Group (EORTC/MSG) Consensus Group Clin Infect Dis 2008 46 1813 1821 10.1086/588660 18462102 PMC2671227

[B17] Guinea J Torres-Narbona M Gijon P Munoz P Pozo F Pelaez T de Miguel J Bouza E Pulmonary aspergillosis in patients with chronic obstructive pulmonary disease: incidence, risk factors, and outcome Clin Microbiol Infect 2010 16 870 877 19906275 10.1111/j.1469-0691.2009.03015.x

[B18] Husain S Paterson DL Studer SM Crespo M Pilewski J Durkin M Wheat JL Johnson B McLaughlin L Bentsen C McCurry KR Singh N *Aspergillus *galactomannan antigen in the bronchoalveolar lavage fluid for the diagnosis of invasive aspergillosis in lung transplant recipients Transplantation 2007 83 1330 1336 10.1097/01.tp.0000263992.41003.33 17519782

[B19] Meersseman W Lagrou K Maertens J Wijngaerden EV Invasive aspergillosis in the intensive care unit Clin Infect Dis 2007 45 205 216 10.1086/518852 17578780

[B20] Mennink-Kersten MA Donnelly JP Verweij PE Detection of circulating galactomannan for the diagnosis and management of invasive aspergillosis Lancet Infect Dis 2004 4 349 357 10.1016/S1473-3099(04)01045-X 15172343

[B21] Stuck AE Minder CE Frey FJ Risk of infectious complications in patients taking glucocorticosteroids Rev Infect Dis 1989 11 954 963 10.1093/clinids/11.6.954 2690289

[B22] Balloy V Huerre M Latge JP Chignard M Differences in patterns of infection and inflammation for corticosteroid treatment and chemotherapy in experimental invasive pulmonary aspergillosis Infect Immun 2005 73 494 503 10.1128/IAI.73.1.494-503.2005 15618189 PMC538925

[B23] Tony TC Robson GD Denning DW Hydrocortisone-enhanced growth of *Aspergillus *spp.: implications for pathogenesis Microbiology 1994 140 2475 2479 10.1099/13500872-140-9-2475 7952197

[B24] Pfeiffer CD Fine JP Safdar N Diagnosis of invasive aspergillosis using a galactomannan assay: a meta-analysis Clin Infect Dis 2006 42 1417 1427 10.1086/503427 16619154

[B25] Perfect J Cox G Lee J Kauffman C de Repentigny L Chapman S Morrison V Pappas P Hiemenz J Stevens D Mycoses Study Group The impact of culture isolation of *Aspergillus *species: A hospital-based survey of aspergillosis Clin Infect Dis 2001 33 1824 1833 10.1086/323900 11692293

[B26] Tanriover MD Metan G Altun B Hascelik G Uzun O False positivity for *Aspergillus *antigenemia related to the administration of piperacillin/tazobactam Eur J Intern Med 2005 16 489 491 10.1016/j.ejim.2005.04.007 16275542

[B27] Meersseman W Lagrou K Maertens J Wilmer A Hermans G Vanderschueren S Spriet I Verbeken E Van Wijngaerden E Galactomannan in bronchoalveolar lavage fluid: a tool for diagnosing aspergillosis in intensive care unit patients Am J Respir Crit Care Med 2008 177 27 34 17885264 10.1164/rccm.200704-606OC

[B28] Bulpa P Dive A Diagnosis of invasive bronchial-pulmonary aspergillosis in patients with chronic obstructive respiratory diseases Crit Care 2011 15 420 author reply 420 10.1186/cc10138 21542895 PMC3219395

[B29] Xu H Li L Huang WJ Wang LX Li WF Yuan WF Invasive pulmonary aspergillosis in patients with chronic obstructive pulmonary disease: a case control study from China Clin Microbiol Infect 2012 18 403 408 10.1111/j.1469-0691.2011.03503.x 22023558

[B30] Ader F Invasive pulmonary aspergillosis in patients with chronic obstructive pulmonary disease: an emerging fungal disease Curr Infect Dis Rep 2010 12 409 416 10.1007/s11908-010-0132-1 21308548

[B31] Aquino VR Nagel F Andreolla HF de-Paris F Xavier MO Goldani LZ Denning DW Pasqualotto AC The performance of real-time PCRr, galactomannan, and fungal culture in the diagnosis of invasive aspergillosis in ventilated patients with chronic obstructive pulmonary disease (COPD) Mycopathologia 2012 174 163 169 10.1007/s11046-012-9531-1 22382738

